# Synthesis, crystal structure and Hirshfeld surface analysis of bis­(2-amino-1,3,4-thia­diazol-3-ium) diaqua­dichlorido(propanedioato-κ^2^*O*^1^,*O*^3^)manganate(II)

**DOI:** 10.1107/S205698902600112X

**Published:** 2026-02-13

**Authors:** Guzal Nuralieva, Mushtari Alieva, Ekaterina Kinshakova, Aziz Atashov, Jamshid Ashurov, Shakhnoza Kadirova, Batirbay Torambetov

**Affiliations:** a National University of Uzbekistan named after Mirzo Ulugbek 4 University St Tashkent 100174 Uzbekistan; b Tashkent Pharmaceutical Institute, 45 A. Aybek St. Tashkent, 100015, Uzbekistan; c Karakalpak State University, 1 Ch.Abdirov St. Nukus, 230112, Uzbekistan; d Institute of Bioorganic Chemistry, Academy of Sciences of Uzbekistan, M. Ulugbek St, 83, Tashkent, 100125, Uzbekistan; Vienna University of Technology, Austria

**Keywords:** crystal structure, manganese(II) complex, malonato ligand, thia­diazo­lium cation, hydrogen bonding, supra­mol­ecular inter­actions

## Abstract

In the title salt, (C_2_H_4_N_3_S)_2_[MnCl_2_(C_3_H_2_O_4_)(H_2_O)_2_], the central Mn^II^ atom of the complex anion adopts a distorted octa­hedral coordination environment, defined by two aqua, two chlorido, and one bidentate malonato ligands. The anion is charge balanced by two thia­diazole moieties protonated at one of the heterocyclic N atoms. In the crystal, the cations and anions engage in extensive hydrogen-bonding inter­actions and short S⋯Cl contacts; additional π–π stacking inter­actions are present between adjacent cations. Hirshfeld surface analysis was used to qu­antify the inter­molecular inter­actions of the complex anion, revealing that H⋯O, H⋯Cl, and H⋯H inter­actions contribute most to the crystal packing.

## Abstract 2

In the complex anion of the title salt, the central Mn^II^ atom adopts a distorted octa­hedral coordination environment, defined by two aqua, two chlorido, and one bidentate malonato ligand. The thia­diazole ligands are protonated and linked to the anion through various hydrogen-bonding inter­actions.

## 1. Chemical context

The 1,3,4-thia­diazole ring is a five-membered aromatic heterocycle with different isomeric forms (1,2,3-thia­diazole, 1,2,4-thia­diazole, 1,2,5-thia­diazole, and 1,3,4-thia­diazole). The 1,3,4-isomer is the most extensively studied due to its wide range of biological and pharmacological activities, including anti­microbial, anti­fungal, anti­tubercular, anti-inflammatory, anti­convulsant, anti­oxidant, anti­hypertensive, and anti­cancer effects (Ahmad *et al.*, 2024 ▸; Parmar & Umrigar, 2017 ▸; Hu *et al.*, 2014 ▸; Kinshakova *et al.*, 2025 ▸; Chou *et al.*, 2003 ▸). Additionally, the N–C–S moiety within the 1,3,4-thia­diazole ring enables strong coordination with metal ions through its nitro­gen and sulfur donor atoms, forming stable metal complexes (Lynch, 2002 ▸; Zhu *et al.*, 2017 ▸; Kadirova *et al.*, 2022 ▸; Atashov *et al.*, 2024 ▸). This combination of biological efficacy and coordination versatility underscores its importance in both medicinal and coordination chemistry. Several metal complexes are known to exhibit enhanced biological activities compared to the control or parent ligand/drug after complex formation (Femi & Ayoola, 2012 ▸). In particular, manganese complexes have been shown to possess promising biological activities; however, they remain relatively underexplored and insufficiently studied (Kozieł *et al.*, 2024 ▸).

In this article, we report on a salt consisting of cations from a 1,3,4-thia­diazole derivative and a manganese-based anion containing aqua, chlorido and malonato ligands, (*L*H)_2_[MnCl_2_(C_3_H_2_O_4_)(H_2_O)_2_] (*L* is 2-amino-1,3,4-thia­diazole, C_2_H_3_N_3_S).
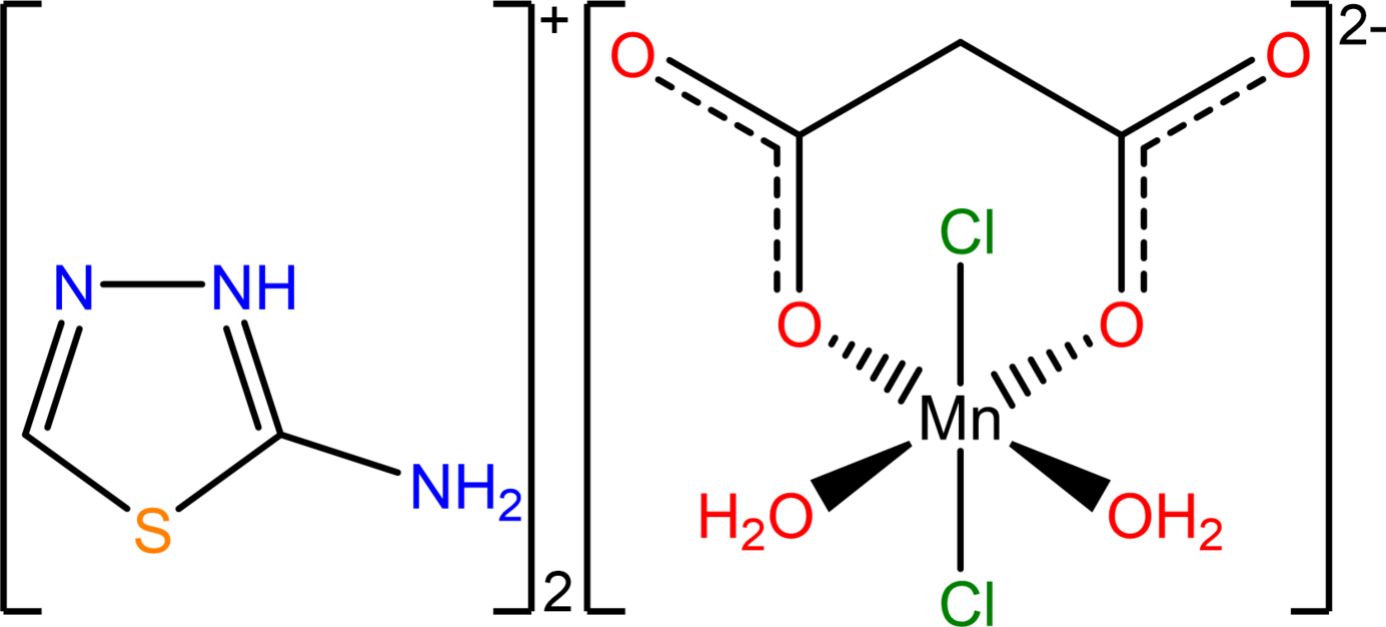


## 2. Structural commentary

**Figure 1:**
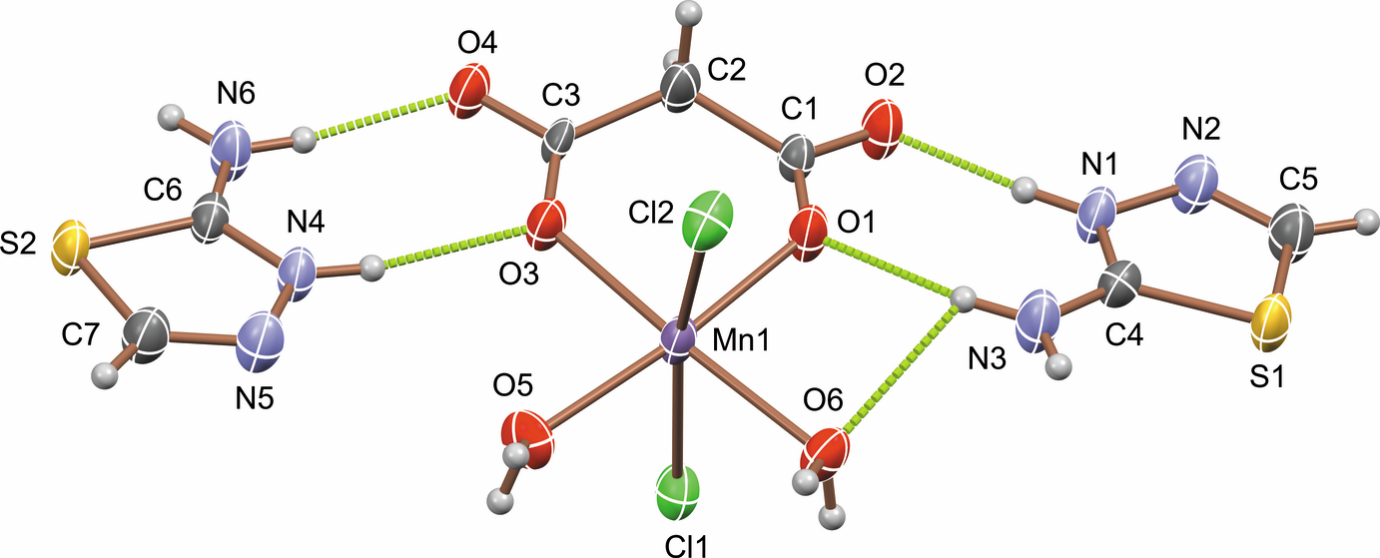
The asymmetric unit of (*L*H)_2_[Mn(H_2_O)_2_Cl_2_(C_3_O_4_H_2_)] drawn with displacement ellipsoids at the 50% probability level; hydrogen atoms are displayed as small spheres of arbitrary size. Inter­molecular hydrogen-bonding inter­actions are shown as green dashed lines.

The asymmetric unit of (*L*H)_2_[Mn(H_2_O)_2_Cl_2_(C_3_O_4_H_2_)] comprises two (*L*H)^+^ cations, both protonated on the N atom at the 3-position of the heterocycle (atom numbering N1 and N4), and one [Mn(H_2_O)_2_Cl_2_(C_3_O_4_H_2_)]^2–^ dianion (Fig. 1 ▸). The central manganese(II) atom is distorted octa­hedrally coordinated by two aqua ligands, two chlorido ligands, and one malonato ligand, which acts as a bidentate ligand through two of its carboxyl­ate oxygen atoms. The two chlorido ligands are situated *trans* to each other at the axial positions, while the equatorial plane is defined by the bidentate malonato ligand and the two *cis*-positioned aqua ligands. The Mn—O bond lengths range from 2.136 (3) to 2.194 (3) Å, while the Mn—Cl bonds are significantly longer [2.5363 (14) to 2.6243 (13) Å]. From the coordination environment and charge balance, the oxidation state of the manganese ion is determined to be +II.

## 3. Supra­molecular features

**Figure 2:**
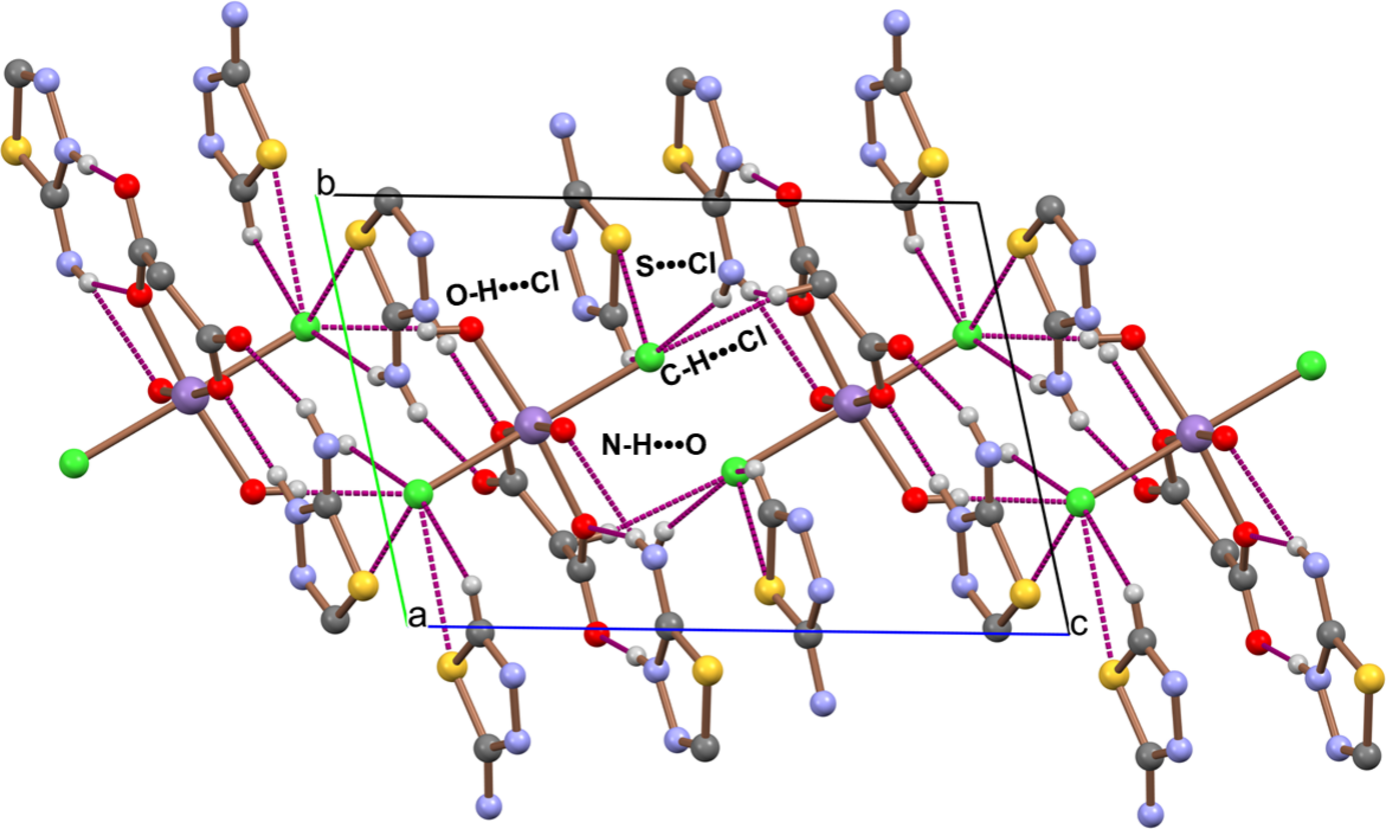
A view of the crystal packing of mol­ecules along the *a* axis in the crystal structure of (*L*H)_2_[Mn(H_2_O)_2_Cl_2_(C_3_O_4_H_2_)] including the most important hydrogen-bonding inter­actions as colored dashed lines.

(*L*H)_2_[Mn(H_2_O)_2_Cl_2_(C_3_O_4_H_2_)] exhibits an intricate network of inter­molecular inter­actions due to the presence of multiple hydrogen-bonding sites. These include N—H⋯O, N—H⋯Cl, and C—H⋯Cl inter­actions with the donor sites located at the two thia­diazo­lium cations, and with O—H⋯O, O—H⋯N and O—H⋯Cl inter­actions of the aqua ligands. Numerical details are given in Table 1 ▸. In addition, short S⋯Cl contacts between the cations and anion are present, ranging from 3.1867 (18) to 3.4457 (17) Å, shorter than the sum of the van der Waals radii for S and Cl (≈3.55 Å; Bondi, 1964 ▸), as well as a π–π stacking inter­action between two adjacent thia­diazo­lium cations, with a centroid-to-centroid distance of 3.620 (3) Å (slippage: 1.338 Å, *Cg*2*⋯g*3(–1 + *x*, −1 + *y*, *z*); *Cg*2 and *Cg*3 are the centroids of the S1–C4–N1–N2–C5 and S2–C6–N4–N5–C7 rings, respectively). The packing of the mol­ecules is shown in Fig. 2 ▸.

## 4. Hirshfeld surface analysis

Hirshfeld surface (Spackman & Jayatilaka, 2009 ▸) and two-dimensional fingerprint plot analyses (Spackman & McKinnon, 2002 ▸) were performed using the program *CrystalExplorer* (Spackman *et al.*, 2021 ▸) to investigate and qu­antify the inter­mol­ecular inter­actions responsible for the consolidation of the crystal packing. For the sake of clarity, only inter­actions of the complex anion were considered.

**Figure 3:**
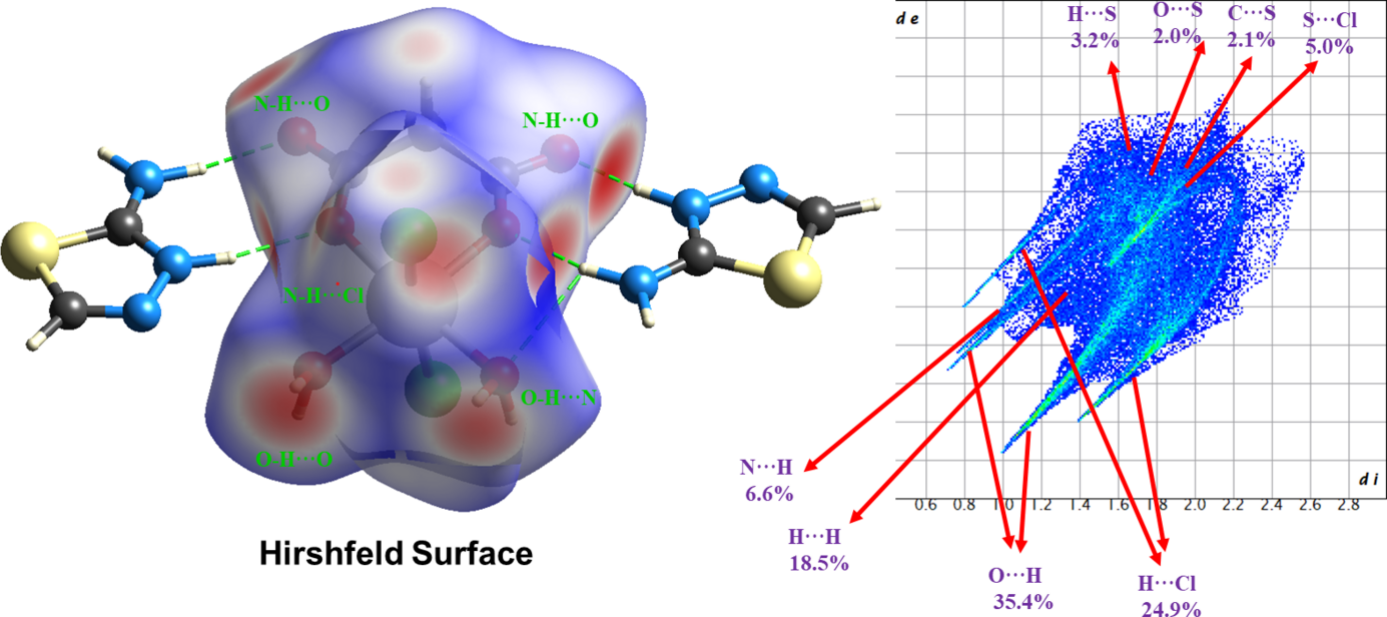
(Left) Hirshfeld surface of the [Mn(H_2_O)_2_Cl_2_(C_3_O_4_H_2_)]^2–^ anion within the crystal structure of (*L*H)_2_[Mn(H_2_O)_2_Cl_2_(C_3_O_4_H_2_)]; (right) two-dimensional fingerprint plot showing the contributions of different inter­molecular contacts to the overall Hirshfeld surface area.

As expected, the Hirshfeld surface (HS) of [Mn(H_2_O)_2_Cl_2_(C_3_O_4_H_2_)] displays several prominent dark-red spots indicative of significant inter­molecular inter­actions. A pair of red regions on opposite sides of the surface correspond to close N—H⋯O hydrogen-bonding inter­actions. Additionally, two red spots on the front side of the HS indicate short O—H⋯O and O—H⋯N contacts. A distinct red spot near the chloride atom suggests the presence of an N—H⋯Cl inter­action (Fig. 3 ▸, left). The major inter­mol­ecular inter­actions contributing to the Hirshfeld surface area (97.7%) are visualized through the two-dimensional fingerprint plots (Fig. 3 ▸, right). These include O⋯H (35.4%), H⋯Cl (24.9%), H⋯H (18.5%), N⋯H (6.6%), S⋯Cl (5.0%), H⋯S (3.2%), C⋯S (2.1%), and O⋯S (2.0%) contacts. Minor inter­actions C⋯O (0.8%), C⋯H (0.6%), C⋯C (0.3%), and N⋯O (0.5%) collectively contribute less than 3% to the total HS area of [Mn(H_2_O)_2_Cl_2_(C_3_O_4_H_2_)].

## 5. Database survey

A database survey conducted using the ConQuest program within the Cambridge Structural Database (CSD, Version 6.00, March 2025; Groom *et al.*, 2016 ▸) identified eight crystal structures containing metals such as cobalt, copper, and zinc, in which the ligand (*L*) coordinates monodentately to the metal cations *via* the endocyclic nitro­gen atom at the 3-position (CSD refcode FICCOJ, Ishankhodzhaeva *et al.*, 1998 ▸; GAGVIV, GAGVOB, Wang *et al.*, 2010 ▸; GOKXOT, Khusenov *et al.*, 1998 ▸; NIYDII, Khusenov *et al.*, 1997 ▸; ZEKWOE, Gurbanov *et al.*, 2018 ▸; FUXKIW; Nuralieva *et al.*, 2025 ▸; JOJLUT, Kadirova *et al.*, 2022 ▸). Additionally, two structures were found where *L* acts as a bridging bidentate ligand, coordinating through two endocyclic nitro­gen atoms to copper and silver cations (LIXSEQ, LIXSAM, Maekawa *et al.*, 1999 ▸). Furthermore, two complexes involving bis­muth and anti­mony were reported where the nitro­gen atom at the 3-position is protonated, resulting in non-coordinating thia­diazo­lium cations (GIKBIL, Antolini *et al.*, 1988 ▸; LEYHEC, Cornia *et al.*, 1994 ▸). Notably, no crystal structures have been reported so far in which a complex manganate anion is charge-balanced by a thia­diazo­lium cation.

## 6. Synthesis and crystallization

A solution of malonic acid (0.0052 g, 0.05 mmol) in 3 ml of ethanol was neutralized with sodium hydroxide (0.0052 g, 0.13 mmol) in 3 ml of ethanol and the mixture was heated at 323 K for 1 h under stirring. Separately, MnCl_2_·4H_2_O (0.099 g, 0.5 mmol) was dissolved in 3 ml of water, and 2-amino-1,3,4-thia­diazole (*L*) (0.101 g, 1 mmol) was dissolved in 3 ml of ethanol. The ligand solution was added dropwise to the MnCl_2_·4H_2_O solution under stirring, followed by the addition of the sodium malonate solution. Single crystals of the title complex, suitable for X-ray diffraction analysis, were obtained by slow evaporation of the solvent over 5 d. Yield: 78%, m.p. 493 K.

## 7. Refinement

Crystal data, data collection and structure refinement details are summarized in Table 2 ▸. All hydrogen atoms were located from difference-Fourier maps and refined with a riding model; DFIX restraints were applied to some of the O—H bond lengths.

## Supplementary Material

Crystal structure: contains datablock(s) I. DOI: 10.1107/S205698902600112X/wm5781sup1.cif

Structure factors: contains datablock(s) I. DOI: 10.1107/S205698902600112X/wm5781Isup3.hkl

CCDC reference: 2495418

Additional supporting information:  crystallographic information; 3D view; checkCIF report

## Figures and Tables

**Table 1:** Hydrogen-bond geometry (Å, °)

*D*—H⋯*A*	*D*—H	H⋯*A*	*D*⋯*A*	*D*—H⋯*A*
O5—H5*A*⋯O2^i^	0.85 (1)	1.93 (2)	2.761 (5)	166 (5)
O5—H5*B*⋯Cl1^ii^	0.85 (1)	2.34 (1)	3.186 (4)	175 (7)
O6—H6*A*⋯O4^iii^	0.85	2.08	2.853 (5)	151
O6—H6*B*⋯N2^i^	0.85	2.06	2.866 (5)	158
N1—H1⋯O2	0.86	1.78	2.641 (5)	175
N3—H3*A*⋯O1	0.86	2.02	2.854 (5)	162
N3—H3*B*⋯Cl2^iv^	0.86	2.39	3.207 (5)	158
N4—H4⋯O3	0.86	2.07	2.919 (5)	171
N6—H6*C*⋯O4	0.86	1.92	2.760 (5)	165
N6—H6*D*⋯Cl1^v^	0.86	2.45	3.243 (4)	154
N6—H6*D*⋯Cl1^vi^	0.86	2.99	3.485 (4)	118
C2—H2*A*⋯N5^vii^	0.97	2.55	3.487 (6)	162
C2—H2*B*⋯Cl2^viii^	0.97	2.79	3.741 (6)	166
C5—H5⋯Cl2^ix^	0.93	2.98	3.742 (5)	140
C5—H5⋯Cl2^x^	0.93	2.90	3.344 (5)	111
C7—H7⋯Cl1^xi^	0.93	2.94	3.438 (5)	115
C7—H7⋯O4^i^	0.93	2.42	3.277 (6)	154

Symmetry codes: (i) 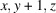; (ii) 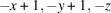; (iii) 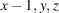; (iv) 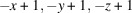; (v) 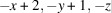; (vi) 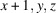; (vii) 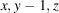; (viii) 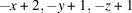; (ix) 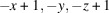; (x) 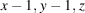; (xi) 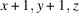.

**Table 2:** Experimental details

Crystal data	
Chemical formula	(C_2_H_4_N_3_S)_2_[MnCl_2_(C_3_H_2_O_4_)(H_2_O)_2_]
*M*_r_	468.20
Crystal system, space group	Triclinic, *P* 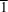
Temperature (K)	293
*a*, *b*, *c* (Å)	8.4382 (3), 8.6103 (3), 12.6557 (4)
α, β, γ (°)	98.510 (2), 101.845 (3), 106.552 (3)
*V* (Å^3^)	841.49 (5)
*Z*	2
Radiation type	Cu *K*α
μ (mm^−1^)	12.01
Crystal size (mm)	0.16 × 0.14 × 0.09

Data collection	
Diffractometer	XtaLAB Synergy, Single source at home/near, HyPix3000
Absorption correction	Multi-scan (*CrysAlis PRO*; Rigaku OD, 2023 ▸)
*T*_min_, *T*_max_	0.282, 1.000
No. of measured, independent and observed [*I* > 2σ(*I*)] reflections	7249, 3240, 2690
*R*_int_	0.071
(sin θ/λ)_max_ (Å^−1^)	0.615

Refinement	
*R*[*F*^2^ > 2σ(*F*^2^)], *wR*(*F*^2^), *S*	0.069, 0.192, 1.02
No. of reflections	3240
No. of parameters	226
No. of restraints	4
H-atom treatment	H atoms treated by a mixture of independent and constrained refinement
Δρ_max_, Δρ_min_ (e Å^−3^)	1.33, −1.16

Computer programs: *CrysAlis PRO* (Rigaku OD, 2023 ▸), *SHELXT* (Sheldrick, 2015*a* ▸), *SHELXL* (Sheldrick, 2015*b* ▸) and *OLEX2* (Dolomanov *et al.*, 2009 ▸ ▸).

## supplementary crystallographic information

### (I) Crystal data

**Table:**

(C_2_H_4_N_3_S)_2_[MnCl_2_(C_3_H_2_O_4_)(H_2_O)_2_]	*Z* = 2
*M**_r_* = 468.20	*F*(000) = 474
Triclinic, *P*1	*D*_x_ = 1.848 Mg m^−^^3^
*a* = 8.4382 (3) Å	Cu *K*α radiation, λ = 1.54184 Å
*b* = 8.6103 (3) Å	Cell parameters from 4604 reflections
*c* = 12.6557 (4) Å	θ = 3.7–71.7°
α = 98.510 (2)°	µ = 12.01 mm^−^^1^
β = 101.845 (3)°	*T* = 293 K
γ = 106.552 (3)°	Block, colourless
*V* = 841.49 (5) Å^3^	0.16 × 0.14 × 0.09 mm

### (I) Data collection

**Table:**

XtaLAB Synergy, Single source at home/near, HyPix3000 diffractometer	2690 reflections with *I* > 2σ(*I*)
Detector resolution: 10.0000 pixels mm^-1^	*R*_int_ = 0.071
ω scans	θ_max_ = 71.5°, θ_min_ = 3.7°
Absorption correction: multi-scan (CrysAlis PRO; Rigaku OD, 2023)	*h* = −9→10
*T*_min_ = 0.282, *T*_max_ = 1.000	*k* = −10→10
7249 measured reflections	*l* = −15→15
3240 independent reflections	

### (I) Refinement

**Table:**

Refinement on *F*^2^	4 restraints
Least-squares matrix: full	Hydrogen site location: mixed
*R*[*F*^2^ > 2σ(*F*^2^)] = 0.069	H atoms treated by a mixture of independent and constrained refinement
*wR*(*F*^2^) = 0.192	*w* = 1/[σ^2^(*F*_o_^2^) + (0.1342*P*)^2^] where *P* = (*F*_o_^2^ + 2*F*_c_^2^)/3
*S* = 1.02	(Δ/σ)_max_ < 0.001
3240 reflections	Δρ_max_ = 1.33 e Å^−^^3^
226 parameters	Δρ_min_ = −1.15 e Å^−^^3^

### (I) Special details

**Table:**

Geometry. All esds (except the esd in the dihedral angle between two l.s. planes) are estimated using the full covariance matrix. The cell esds are taken into account individually in the estimation of esds in distances, angles and torsion angles; correlations between esds in cell parameters are only used when they are defined by crystal symmetry. An approximate (isotropic) treatment of cell esds is used for estimating esds involving l.s. planes.

### (I) Fractional atomic coordinates and isotropic or equivalent isotropic displacement parameters (Å^2^)

**Table:**

	*x*	*y*	*z*	*U*_iso_*/*U*_eq_	
Mn1	0.66473 (8)	0.47031 (8)	0.25494 (6)	0.0279 (3)	
Cl1	0.46492 (14)	0.30744 (13)	0.05857 (10)	0.0351 (3)	
Cl2	0.80574 (14)	0.63155 (15)	0.45305 (10)	0.0411 (3)	
S1	0.14252 (15)	−0.09459 (14)	0.43995 (11)	0.0375 (3)	
S2	1.29985 (14)	0.90727 (13)	0.05433 (10)	0.0341 (3)	
O1	0.6478 (4)	0.2375 (4)	0.2996 (3)	0.0365 (8)	
O3	0.8909 (4)	0.4502 (4)	0.2069 (3)	0.0323 (7)	
O6	0.4238 (4)	0.4601 (4)	0.2983 (3)	0.0359 (8)	
H6A	0.341222	0.420929	0.240211	0.054*	
H6B	0.424141	0.558262	0.320859	0.054*	
O4	1.0798 (4)	0.3374 (4)	0.1635 (3)	0.0364 (8)	
O2	0.6671 (4)	−0.0131 (4)	0.2839 (3)	0.0377 (8)	
O5	0.7029 (5)	0.6919 (4)	0.1910 (3)	0.0384 (8)	
N4	1.0705 (5)	0.7358 (5)	0.1291 (3)	0.0320 (8)	
H4	1.007128	0.654413	0.149434	0.038*	
N1	0.3951 (5)	−0.0948 (5)	0.3625 (4)	0.0353 (9)	
H1	0.482529	−0.062812	0.336449	0.042*	
N2	0.3283 (5)	−0.2566 (5)	0.3715 (4)	0.0404 (10)	
N6	1.2331 (5)	0.5837 (5)	0.0649 (4)	0.0394 (10)	
H6C	1.176063	0.496823	0.083398	0.047*	
H6D	1.314917	0.580369	0.034650	0.047*	
N3	0.3647 (5)	0.1676 (5)	0.3959 (4)	0.0450 (11)	
H3A	0.452465	0.211237	0.372695	0.054*	
H3B	0.308031	0.228033	0.419026	0.054*	
N5	1.0480 (5)	0.8885 (5)	0.1429 (4)	0.0403 (10)	
C1	0.7247 (5)	0.1384 (5)	0.2811 (4)	0.0247 (8)	
C3	0.9570 (5)	0.3379 (5)	0.2064 (4)	0.0229 (8)	
C4	0.3170 (6)	0.0078 (6)	0.3961 (4)	0.0315 (10)	
C6	1.1961 (5)	0.7190 (5)	0.0824 (4)	0.0295 (9)	
C2	0.9015 (6)	0.1940 (6)	0.2619 (5)	0.0356 (11)	
H2A	0.915146	0.098177	0.218655	0.043*	
H2B	0.983146	0.221204	0.333611	0.043*	
C7	1.1577 (6)	0.9867 (6)	0.1076 (4)	0.0381 (11)	
H7	1.161906	1.096334	0.109999	0.046*	
C5	0.1976 (6)	−0.2732 (6)	0.4114 (5)	0.0397 (11)	
H5	0.136561	−0.373942	0.424126	0.048*	
H5A	0.710 (7)	0.787 (3)	0.226 (4)	0.041 (15)*	
H5B	0.653 (8)	0.687 (9)	0.124 (2)	0.07 (2)*	

### (I) Atomic displacement parameters (Å^2^)

**Table:**

	*U*^11^	*U*^22^	*U*^33^	*U*^12^	*U*^13^	*U*^23^
Mn1	0.0236 (4)	0.0234 (4)	0.0443 (5)	0.0095 (3)	0.0184 (3)	0.0132 (3)
Cl1	0.0338 (6)	0.0290 (5)	0.0421 (7)	0.0051 (4)	0.0149 (5)	0.0099 (4)
Cl2	0.0329 (6)	0.0433 (6)	0.0445 (7)	0.0068 (5)	0.0157 (5)	0.0054 (5)
S1	0.0318 (6)	0.0388 (6)	0.0507 (8)	0.0115 (5)	0.0252 (5)	0.0168 (5)
S2	0.0293 (6)	0.0267 (5)	0.0494 (7)	0.0046 (4)	0.0185 (5)	0.0152 (5)
O1	0.0334 (17)	0.0294 (15)	0.062 (2)	0.0152 (13)	0.0291 (16)	0.0220 (15)
O3	0.0268 (15)	0.0286 (15)	0.051 (2)	0.0116 (12)	0.0214 (14)	0.0152 (14)
O6	0.0265 (16)	0.0317 (16)	0.052 (2)	0.0099 (13)	0.0160 (15)	0.0075 (14)
O4	0.0287 (16)	0.0332 (16)	0.058 (2)	0.0128 (13)	0.0246 (16)	0.0179 (15)
O2	0.0364 (18)	0.0256 (15)	0.061 (2)	0.0115 (13)	0.0274 (16)	0.0164 (14)
O5	0.050 (2)	0.0210 (15)	0.047 (2)	0.0128 (14)	0.0154 (18)	0.0101 (14)
N4	0.0280 (19)	0.0298 (18)	0.041 (2)	0.0056 (15)	0.0176 (17)	0.0121 (16)
N1	0.0266 (19)	0.034 (2)	0.048 (2)	0.0059 (16)	0.0197 (17)	0.0133 (17)
N2	0.033 (2)	0.0317 (19)	0.060 (3)	0.0081 (16)	0.020 (2)	0.0143 (19)
N6	0.038 (2)	0.0294 (19)	0.062 (3)	0.0119 (16)	0.028 (2)	0.0205 (19)
N3	0.039 (2)	0.036 (2)	0.071 (3)	0.0122 (18)	0.030 (2)	0.023 (2)
N5	0.038 (2)	0.031 (2)	0.059 (3)	0.0123 (17)	0.024 (2)	0.0124 (19)
C1	0.0233 (19)	0.0191 (18)	0.032 (2)	0.0033 (15)	0.0109 (17)	0.0068 (15)
C3	0.0144 (17)	0.0203 (17)	0.033 (2)	0.0007 (14)	0.0106 (16)	0.0062 (15)
C4	0.024 (2)	0.036 (2)	0.037 (3)	0.0092 (18)	0.0121 (18)	0.0112 (19)
C6	0.024 (2)	0.031 (2)	0.031 (2)	0.0023 (17)	0.0103 (18)	0.0081 (17)
C2	0.029 (2)	0.037 (2)	0.057 (3)	0.0173 (19)	0.025 (2)	0.024 (2)
C7	0.034 (2)	0.035 (2)	0.051 (3)	0.012 (2)	0.019 (2)	0.013 (2)
C5	0.036 (3)	0.032 (2)	0.054 (3)	0.007 (2)	0.019 (2)	0.014 (2)

### (I) Geometric parameters (Å, º)

**Table:**

Mn1—Cl1	2.6243 (13)	N4—N5	1.372 (5)
Mn1—Cl2	2.5363 (14)	N4—C6	1.347 (6)
Mn1—O1	2.136 (3)	N1—H1	0.8600
Mn1—O3	2.162 (3)	N1—N2	1.378 (5)
Mn1—O6	2.194 (3)	N1—C4	1.316 (6)
Mn1—O5	2.148 (3)	N2—C5	1.285 (7)
S1—C4	1.730 (4)	N6—H6C	0.8600
S1—C5	1.739 (5)	N6—H6D	0.8600
S2—C6	1.735 (4)	N6—C6	1.289 (6)
S2—C7	1.739 (5)	N3—H3A	0.8600
O1—C1	1.234 (5)	N3—H3B	0.8600
O3—C3	1.246 (5)	N3—C4	1.319 (6)
O6—H6A	0.8501	N5—C7	1.268 (6)
O6—H6B	0.8502	C1—C2	1.513 (6)
O4—C3	1.265 (5)	C3—C2	1.520 (6)
O2—C1	1.264 (5)	C2—H2A	0.9700
O5—H5A	0.850 (10)	C2—H2B	0.9700
O5—H5B	0.851 (10)	C7—H7	0.9300
N4—H4	0.8600	C5—H5	0.9300
			
Cl2—Mn1—Cl1	169.11 (5)	H6C—N6—H6D	120.0
O1—Mn1—Cl1	88.68 (11)	C6—N6—H6C	120.0
O1—Mn1—Cl2	92.49 (11)	C6—N6—H6D	120.0
O1—Mn1—O3	84.92 (12)	H3A—N3—H3B	120.0
O1—Mn1—O6	89.57 (12)	C4—N3—H3A	120.0
O1—Mn1—O5	170.65 (14)	C4—N3—H3B	120.0
O3—Mn1—Cl1	92.14 (10)	C7—N5—N4	109.2 (4)
O3—Mn1—Cl2	98.75 (10)	O1—C1—O2	122.8 (4)
O3—Mn1—O6	173.54 (11)	O1—C1—C2	121.6 (3)
O6—Mn1—Cl1	84.35 (10)	O2—C1—C2	115.4 (4)
O6—Mn1—Cl2	84.83 (10)	O3—C3—O4	123.0 (4)
O5—Mn1—Cl1	87.40 (11)	O3—C3—C2	122.4 (4)
O5—Mn1—Cl2	92.96 (11)	O4—C3—C2	114.5 (3)
O5—Mn1—O3	86.75 (13)	N1—C4—S1	111.1 (3)
O5—Mn1—O6	98.48 (14)	N1—C4—N3	124.6 (4)
C4—S1—C5	87.3 (2)	N3—C4—S1	124.3 (4)
C6—S2—C7	87.5 (2)	N4—C6—S2	109.2 (3)
C1—O1—Mn1	131.3 (3)	N6—C6—S2	126.6 (4)
C3—O3—Mn1	131.2 (3)	N6—C6—N4	124.2 (4)
Mn1—O6—H6A	109.4	C1—C2—C3	121.6 (4)
Mn1—O6—H6B	109.4	C1—C2—H2A	106.9
H6A—O6—H6B	104.5	C1—C2—H2B	106.9
Mn1—O5—H5A	125 (4)	C3—C2—H2A	106.9
Mn1—O5—H5B	120 (5)	C3—C2—H2B	106.9
H5A—O5—H5B	105 (6)	H2A—C2—H2B	106.7
N5—N4—H4	121.4	S2—C7—H7	121.5
C6—N4—H4	121.4	N5—C7—S2	117.0 (4)
C6—N4—N5	117.1 (4)	N5—C7—H7	121.5
N2—N1—H1	122.0	S1—C5—H5	122.2
C4—N1—H1	122.0	N2—C5—S1	115.5 (4)
C4—N1—N2	115.9 (4)	N2—C5—H5	122.2
C5—N2—N1	110.1 (4)		
			
Mn1—O1—C1—O2	−158.3 (4)	N2—N1—C4—N3	178.5 (5)
Mn1—O1—C1—C2	26.3 (7)	N5—N4—C6—S2	1.1 (5)
Mn1—O3—C3—O4	171.6 (3)	N5—N4—C6—N6	179.5 (5)
Mn1—O3—C3—C2	−11.2 (7)	C4—S1—C5—N2	−1.4 (5)
O1—C1—C2—C3	−32.3 (7)	C4—N1—N2—C5	0.7 (7)
O3—C3—C2—C1	24.6 (7)	C6—S2—C7—N5	0.6 (5)
O4—C3—C2—C1	−158.0 (4)	C6—N4—N5—C7	−0.6 (6)
O2—C1—C2—C3	152.0 (5)	C7—S2—C6—N4	−0.9 (4)
N4—N5—C7—S2	−0.2 (6)	C7—S2—C6—N6	−179.3 (5)
N1—N2—C5—S1	0.7 (6)	C5—S1—C4—N1	1.7 (4)
N2—N1—C4—S1	−1.8 (6)	C5—S1—C4—N3	−178.6 (5)

### (I) Hydrogen-bond geometry (Å, º)

**Table:**

*D*—H···*A*	*D*—H	H···*A*	*D*···*A*	*D*—H···*A*
O5—H5*A*···O2^i^	0.85 (1)	1.93 (2)	2.761 (5)	166 (5)
O5—H5*B*···Cl1^ii^	0.85 (1)	2.34 (1)	3.186 (4)	175 (7)
O6—H6*A*···O4^iii^	0.85	2.08	2.853 (5)	151
O6—H6*B*···N2^i^	0.85	2.06	2.866 (5)	158
N1—H1···O2	0.86	1.78	2.641 (5)	175
N3—H3*A*···O1	0.86	2.02	2.854 (5)	162
N3—H3*B*···Cl2^iv^	0.86	2.39	3.207 (5)	158
N4—H4···O3	0.86	2.07	2.919 (5)	171
N6—H6*C*···O4	0.86	1.92	2.760 (5)	165
N6—H6*D*···Cl1^v^	0.86	2.45	3.243 (4)	154
N6—H6*D*···Cl1^vi^	0.86	2.99	3.485 (4)	118
C2—H2*A*···N5^vii^	0.97	2.55	3.487 (6)	162
C2—H2*B*···Cl2^viii^	0.97	2.79	3.741 (6)	166
C5—H5···Cl2^ix^	0.93	2.98	3.742 (5)	140
C5—H5···Cl2^x^	0.93	2.90	3.344 (5)	111
C7—H7···Cl1^xi^	0.93	2.94	3.438 (5)	115
C7—H7···O4^i^	0.93	2.42	3.277 (6)	154

Symmetry codes: (i) *x*, *y*+1, *z*; (ii) −*x*+1, −*y*+1, −*z*; (iii) *x*−1, *y*, *z*; (iv) −*x*+1, −*y*+1, −*z*+1; (v) −*x*+2, −*y*+1, −*z*; (vi) *x*+1, *y*, *z*; (vii) *x*, *y*−1, *z*; (viii) −*x*+2, −*y*+1, −*z*+1; (ix) −*x*+1, −*y*, −*z*+1; (x) *x*−1, *y*−1, *z*; (xi) *x*+1, *y*+1, *z*.
